# Genomic Analysis of γ-Hexachlorocyclohexane-Degrading *Sphingopyxis lindanitolerans* WS5A3p Strain in the Context of the Pangenome of *Sphingopyxis*

**DOI:** 10.3390/genes10090688

**Published:** 2019-09-06

**Authors:** Michal A. Kaminski, Adam Sobczak, Andrzej Dziembowski, Leszek Lipinski

**Affiliations:** 1 Institute of Biochemistry and Biophysics, Polish Academy of Sciences, Pawinskiego 5a, 02-106 Warsaw, Poland; 2 Institute of Genetics and Biotechnology, Faculty of Biology, University of Warsaw, Pawinskiego 5a, 02-106 Warsaw, Poland

**Keywords:** *Sphingopyxis lindanitolerans*, pesticide, complete genome sequence, pangenome, γ-HCH degradation, *lin* genes

## Abstract

*Sphingopyxis* inhabit diverse environmental niches, including marine, freshwater, oceans, soil and anthropogenic sites. The genus includes 20 phylogenetically distinct, valid species, but only a few with a sequenced genome. In this work, we analyzed the nearly complete genome of the newly described species, *Sphingopyxis lindanitolerans*, and compared it to the other available *Sphingopyxis* genomes. The genome included 4.3 Mbp in total and consists of a circular chromosome, and two putative plasmids. Among the identified set of *lin* genes responsible for γ-hexachlorocyclohexane pesticide degradation, we discovered a gene coding for a new isoform of the LinA protein. The significant potential of this species in the remediation of contaminated soil is also correlated with the fact that its genome encodes a higher number of enzymes potentially involved in aromatic compound degradation than for most other *Sphingopyxis* strains. Additional analysis of 44 *Sphingopyxis* representatives provides insights into the pangenome of *Sphingopyxis* and revealed a core of 734 protein clusters and between four and 1667 unique proteins per genome.

## 1. Introduction

Intensive agriculture entails the extensive use of pesticides to increase yields. One commonly used insecticide has been γ-hexachlorocyclohexane (γ-HCH, also known as lindane or γ-BHC)—a potential carcinogen that is highly toxic to humans, causing neurological diseases, and endocrine disruption [1]. This hydrophobic substance was classified as a persistent organic pollutant [2] and banned in 2009 under the Stockholm Convention. Although the natural degradation of lindane takes a long time, there are known microorganisms that actively degrade it, and can significantly increase the rate of bioremediation [3,4]. Most of the γ-HCH degrading microorganisms belong to *Sphingomonadaceae* (Alphaproteobacteria phylum)—mainly *Sphingobium* sp., *Novosphingobium* sp. and *Sphingomonas* sp. Data showing the γ-HCH degradation activity for the representatives belonging to the *Sphingopyxis* genus has not been published yet, despite the fact that three strains, *Sphingopyxis flava* R11H, *Sphingopyxis indica* DS15 and *Sphingopyxis terrae* subsp. *ummariensis* DSM 24316, were isolated from the environment contaminated with this pesticide [5,6,7,8].

The main γ-HCH degradation pathway was described in detail for *Sphingobium japonicum* UT26S and *Sphingobium indicum* B90A [9,10,11]. These two microorganisms contain the *lin* genes encoding a set of enzymes responsible for lindane conversion to β-ketoadipate, which is further degraded and utilized in other metabolic pathways. The first and key enzyme from the catabolic pathway is dehydrochlorinase LinA [12]. It catalyzes the transformation of γ-HCH to 1,3,4,6-tetrachloro-1,4-cyclohexadiene (1,4-TCDN) via γ-pentachlorocyclohexane (γ-PCCH) by sequential removal of two chloride atoms [13]. This protein is unique among other known dehydrochlorinases as does not require any cofactors [14,15]. LinA is 154 amino acids long and acts as a trimer, where each protomer forms a cone-shaped α + β barrel fold and D25, H73, and R129 residues were shown to be essential for its activity [16,17]. Subsequently, two chloride atoms are removed from 1,4-TCDN by LinB dehalogenase [18] that converts 1,4-TCDN into 2,5-dichloro-2,5-cyclohaxadiene-1,4-diol (2,5-DDOL). LinB requires only H_2_O as a cofactor, and unlike LinA acts on a broad range of halogenated substrates—also on a very stable β-HCH isomer [19]. The last enzyme from upper degradation pathway is LinC [20] that converts 2,5-DDOL to 2,5-dichlorohydroquinone (2,5-DCHQ). LinC belongs to the short-chain alcohol dehydrogenase family and requires NAD^+^ cofactor. Further reactions from downstream degradation pathway involve further removal of chloride atoms from 2,5-DCHQ to hydroquinone (HQ) by reductive dechlorinase LinD [21] and next ring cleavage by ring cleavage oxygenase LinE [22]. It is also known that 2,5-DCHQ can be transformed directly by LinE or LinEb (enzyme identical to LinE in 53% of amino acids) to maleylacetate (MA) through a yet unknown unstable product [10,11]. Next, MA is converted by maleylacetate reductase LinF, to β-ketoadipate and later LinG; LinH and LinJ proteins (acyl-CoA transferase and thiolase) transform β-ketoadipate to succinyl-CoA and acetyl-CoA [11].

Here, we present a detailed genomic analysis of the γ-HCH degrader—*Sphingopyxis lindanitolerans* WS5A3p [23]. As described previously, WS5A3p was selectively enriched using the γ-HCH as a sole carbon source. The genome of this new microorganism was sequenced and analyzed, leading to the identification of 13 *lin* genes involved in the γ-HCH degradation pathway. Additionally, we provide insights into *Sphingopyxis* based on the analysis of 44 genomes, with a description of the core pangenome and the unique proteins for each representative.

## 2. Materials and Methods

### 2.1. Genome Sequencing and Assembly

For genomic DNA isolation, all steps were performed as described previously [23]. Two libraries were prepared for genome sequencing: (1) a paired-end library with an insert size of 500 bp, (2) a Nextera^®^ Mate Pair library (Illumina Inc., San Diego, CA, USA) with an average insert size of 8 kbp. Sequencing was performed on the Illumina MiSeq platform (Illumina Inc.) with a 300 bp read length resulting in 311,675 and 1,376,608 raw paired reads respectively. Next, a number of bioinformatics tools were used to analyze the raw reads. Adapters from the raw reads were trimmed using Cutadapt [24], and quality filtered with Sickle [25] (quality at least Q30). The mate-paired libraries were processed using NxTrim script v0.4.0 provided by the manufacturer. Assembly was performed using the SPAdes 3.9.1 software [26]. The assembly was manually checked by mapping sequencing reads to obtained contigs using Genious 6.1.6 [27]. This Whole Genome Shotgun project has been deposited at DDBJ/ENA/GenBank under the accession PHFW00000000. The version described in this paper is version PHFW01000000. The genome was annotated using the NCBI Prokaryotic Genome Annotation Pipeline [28]. Genes with signal peptides were identified with SignalP 4.1 [29]. Association of genes and proteins to COG categories was done using COGNIZER software [30] and MicroScope platform [31]. Analysis of secondary metabolite biosynthesis gene clusters was done with antiSMASH 5.0 (https://antismash.secondarymetabolites.org) [32]; prophage sequences in WS5A3p genome were identified with PHASTER (http://phaster.ca/) [33]; and CRISPRCasFinder software (http://crispr.i2bc.paris-saclay.fr) [34] with default parameters was used to identify CRISPR-related sequences.

### 2.2. Average Nucleotide Identity Based on BLAST (ANIb) and GGDC Analyses

The similarity of the sequenced genome of *S. lindanitolerans* WS5A3p to other public genomes of closely related *Sphingopyxis* species [8,23,35,36,37,38,39,40,41,42,43,44] was determined based on the average nucleotide identity using both BLASTn (ANIb) and MUMer (ANIm) algorithms with the help of the pyani Python module [45] and average amino acid identity (AAI), which was calculated using CompareM (available at: https://github.com/dparks1134/CompareM).

### 2.3. Pangenomic Analyses

For pangenomic analyses, nucleotide sequences of *Sphingopyxis* sp. genomes were downloaded from the NCBI database on December 2017. Due to low quality and completeness, genomes retrieved from metagenomes and single-cell sequencing were excluded from the analysis. To unify annotation, open reading frames (ORFs) were predicted for all genomes using Prokka [46].

Phylogenetic analysis of *Sphingopyxis* genomes was performed using Phylophlan [47], and ggtree R package [48] was used to display the results. Phylophlan software identifies 400 ubiquitous genes based on their amino acid content, selects the most discriminative positions in each sequence and concatenates them into a single long sequence which is used for ML tree construction by RAxML [49]. From the Phylophlan generated protein alignment we ran RAxML to generate 500 bootstrap replicates (ML search with PMB substitution matrix, as the best-scoring AA model).

For pangenomic analysis of amino acid sequences obtained from annotated ORFs, all genomes were merged into one set. Next, the clustering process based on sequence identity was performed using CD-HIT [50]. Parameters for CD-HIT were as follows: s parameter (length difference cutoff) = 0.9, and c parameter (sequence identity threshold) = 0.8. Using in-house Python and R scripts processing CD-HIT output (scripts collected into Pangenome Analysis Pipeline (PAPi) available at https://github.com/michkam89/PAPi), we identified clusters that had protein representatives present in all genomes (core pangenome) but also clusters specific for each genome included in the analysis (unique clusters). For the unique clusters of each particular genome, we performed additional blastp alignment against other *Sphingopyxis* genomes to exclude false-positive sequences.

*Sphingopyxis* amino acid sequences obtained from annotated ORFs of the analyzed dataset were also searched for the presence of proteins responsible for aromatic compound degradation. For this purpose, we performed a blastp search of all ORFs against in-house protein database developed by Kato and colleagues [51] filtering reads with parameters: e-value <1 × 10^−5^ and alignment percent identity >50%. This database covers the metabolic paths of aerobic and anaerobic degradation routes of 3CB, phenanthrene, biphenyl, and carbazole (phenanthrene-phthalate-protocatechuate route, biphenyl-benzoate route, carbazole-anthranilate route, naphthalene-salicylate route, catechol 1,2- and 2,3-dioxygenation route, gentisate route, anaerobic polycyclic aromatic hydrocarbon (naphthalene) route, and anaerobic benzoate and toluene route) [51].

Alignment of protein sequences for analyzed Lin proteins was performed using MUSCLE [52], phylogenetic relationship and alignment visualization was done in Geneious [27].

## 3. Results and Discussion

The WS5 soil sample, from which the WS5A3p strain was isolated, had been contaminated with a high concentration of organochlorine pesticides including γ-HCH (lindane), dichlorodiphenyltrichloroethane (DDT) and methoxychlor. WS5A3p was isolated through an 8-week enrichment under constant γ-HCH pressure at 5 mg/L [23]. Its activity was confirmed by the ability to create clear zones on γ-HCH agar plates. Moreover, the decrease of γ-HCH was observed in a liquid medium assay where the presence of small amounts of 2,5-dichlorophenol was identified (Figure S1).

The WS5A3p strain was cultured in LB medium and genomic DNA was isolated and sequenced on the Illumina MiSeq platform (see Materials and Methods section) with genome coverage estimated at 112× [23]. As a result of genomic sequencing and assembly, three DNA scaffolds were obtained. General WS5A3p genome statistics are presented in Table S1. The size of the assembled genome sequence is 4,372,786 bp, and contains 65.1% G + C and is 90.4% coding. The assembly consists of one circular scaffold, representing a single chromosome of 4.14 Mbp with three unsequenced gaps of the total estimated length of 305 nucleotides. Two remaining scaffolds of length 181,517 bp and 42,040 bp having separate partition and transfer modules were classified as plasmids. There are a total of 4184 predicted genes, from which 4,133 are protein-coding and 75.1% have assigned putative function. The WS5A3p genome has 51 RNA genes (three rRNA as one ribosomal operon, 45 tRNA and three ncRNA). Observed substitution of lysine 88 by arginine in S12 protein of the 30S ribosomal subunit (RpsL) suggests natural resistance of this strain to streptomycin, what is considered as a feature of *Sphingomonadaceae* and *Erythrobacteraceae* [43]. The functional categorization of genes into COGs (clusters of orthologous groups) for WS5A3p is presented in Table 1. Further analysis of WS5A3p genome in a context of phage-related sequences using PHASTER [33], revealed four prophage regions: one intact region (score = 110) according to the tool’s completeness core, one questionable (score = 90) and two incomplete regions (score = 60 and 50). The intact region (16.1 kb in length) localized on chromosome sequence shows the highest similarity to genome fragment of *Sphingopyxis granuli* TFA [43]. No CRISPR-related sequences were identified in WS5A3p genome using CRISPRCasFinder application [34]. Like other *Sphingopyxis* genomes, this one contains ectoine synthesis genes *ectABCD*, Aspartate kinase Ask_Ect and Ectoine/proline transporter ProP [43]. Secondary metabolite biosynthesis gene clusters analysis using antiSMASH 5.0 also showed the presence of regions potentially involved in the biosynthesis of carotenoids (one region with 75% similarity to astaxanthin dideoxyglycoside cluster) or natural antimicrobials such as β-lactones (one region), lenthipeptides (one region), lasso peptides (three regions), which may give advantage to *S. lindanitolerans* in an oligotrophic environment.

**Figure 1:**
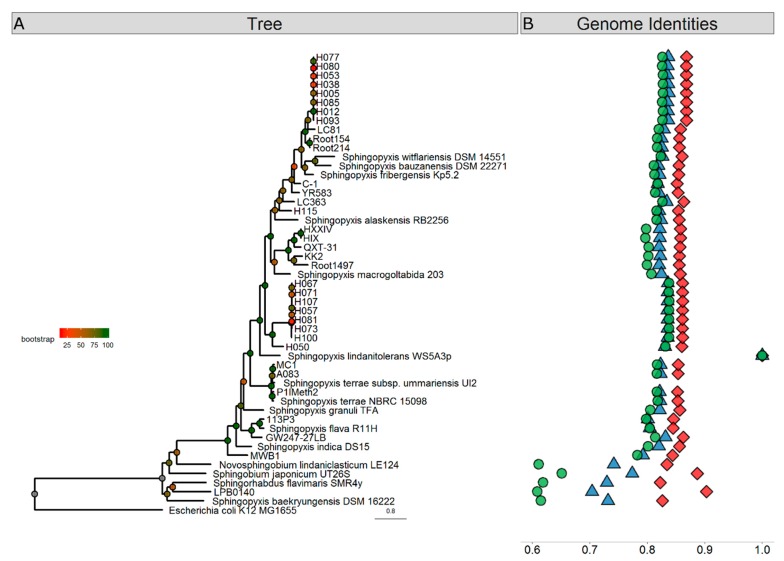
Phylogenetic analyses of *Sphingopyxis* genomes. (**A**) Phylogenetic relationship of *Sphingopyxis* genomes based on a maximum likelihood analysis of 400 conserved protein sequences. Branches are colored according to bootstrap values, which are presented on the nodes. The bar represents 0.8 substitutions per site. The WS5A3p branch is indicated with a blue diamond. The blue shaded box contains outgroup genomes included in the analysis. (**B**) Sequence similarity of analyzed *Sphingopyxis* genomes comparing to WS5A3p strain based on ANIb (blue triangle), ANIm (red diamond) and AAI (green circle).

We compared the WS5A3p genome to 46 other *Sphingopyxis* genomes deposited in the NCBI database. Phylogenetic placement based on 400 highly conserved protein sequences showed that WS5A3p is placed on a distinct branch on the phylogenetic tree with 100% bootstrap support (Figure 1A). We also saw that *Sphingopyxis baekryungensis* DSM 16222 and LPB0140 isolate, previously assigned to *Sphingopyxis,* clustered with outgroup genomes analyzed in the dataset (containing representatives of *Sphingobium, Novosphingobium,* and *Sphingorhabdus*). To extend these findings we also performed genomic analysis of whole DNA sequences (average nucleotide identity based on Mummer—ANIm, average nucleotide identity based on BLAST—ANIb) and amino acid content of all protein sequences (average amino acid Identity—AAI). These tests showed that WS5A3p is unique among available genomes. We also identified that values for MWB1 isolate in all of the three tests were outlying the most from other species, therefore we decided to treat this isolate also as an outgroup. Average values (excluding outgroup and LPB0140, MWB1 and DSM 16222 genomes) for similarity among *Sphingopyxis* genomes to WS5A3p based on ANIb, ANIm and AAI were 82.6 ± 0.98%, 85.8 ± 0.63%, and 81.8 ± 1.35%, respectively. The ranges were 80.1–83.7% for ANIb, 84.5–86.8% for ANIm and 79.7–83.8% for AAI. Results of the tests mentioned above are illustrated in Figure 1B. All of the analyses distinguished two groups of genomes highly similar to each other. Group H1 consisting of isolates: H005, H012, H038, H053, H077, H080, H085, H093 and group H2 consisting of isolates: H057, H067, H071, H073, H081, H100, H107 that are genomic clones with almost identical sequences. This result provides additional evidence that exclusion of *S. baekryungensis* DSM 16222 from *Sphingopyxis* genus and reclassification of isolates LPB0140 and MWB1 should be considered in the near future.

To analyze the uniqueness of WS5A3p genome, we first performed the pangenomic comparison of protein sequences present in all *Sphingopyxis* representatives. From these tests, we excluded genomes of LB0140, MWB1 and DSM 16222, as based on the previous phylogenetic analysis they clustered closely to outgroup genomes (LB0140, DSM 16222) and had AAI values below 80% (LB0140, DSM 16222 and MWB1). Table S2 presents general information about all genomes included in the analysis. Representatives had been isolated from diverse water environments (drinking water, seawater, ground, lake, and cave water) and soils (mainly contaminated with organic pollutants) as well as anthropogenic sites like mines or wastewater treatment plants. Isolates derived from geographically distant regions in Europe, Asia, North America and one from Australia. The median size of the *Sphingopyxis* genome is 4.3 Mbp and ranged from 3.4 Mbp for *Sphingopyxis alaskensis* RB2256 and 5.7 Mbp for *Sphingopyxis macrogoltabida* 203. The average *Sphingopyxis* genome contains 4236 genes with a minimal number of 3287 and maximal of 5572 for *S. alaskensis* RB2256 and *S. macrogoltabida* 203, respectively.

Pangenomic analyses usually focus on pathogenic bacteria at the species level [53]. Few studies have described generic bacterial pangenomes beyond *Streptococcus* [54] or *Bifidobacterium* [55]. The first attempt to describe the *Sphingopyxis* pangenome was performed by Garcia-Romero [33] and Parthasarathy [56]. They analyzed seven *Sphingopyxis* genomes and established the core pangenome size at 1371 gene families or 1515 single copy orthologues. In our pangenomic analysis, we grouped proteins into clusters based on their amino acid sequence, differentiating 734 core clusters present in all of the analyzed genomes, which represent less than 20% of the average *Sphingopyxis* genome. Compared to the previous reports, our dataset increased from seven to 44 genomes and allowed us to reduce the size of the core *Sphingopyxis* pangenome by almost half. It is only 1.5× larger than the minimal genome content of the smallest free-living microorganism (470 coding regions) [57]. We also calculated the size of the entire pangenome with the median size of 28,914 clusters (IQR = 2645). Accumulation plots of the pangenome and core pangenome are presented in Figure S2. While core genome seems to reach its saturation and addition of new genomes should not affect it significantly, the pangenome median curve started to flatten but it is not saturated yet and the pangenome still should be considered as “open”.

**Figure 2:**
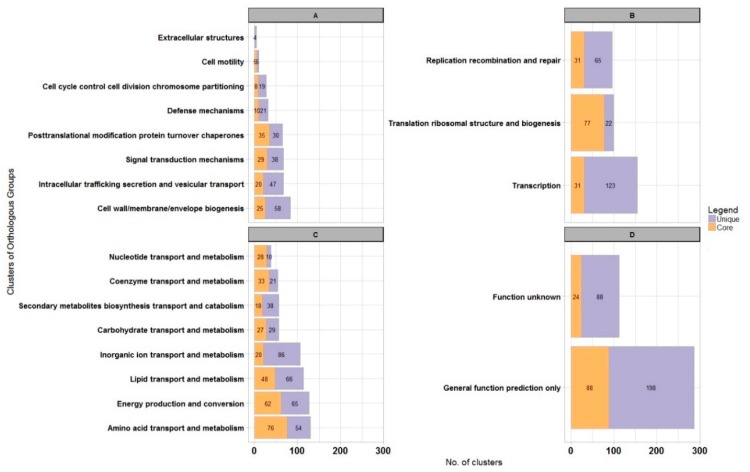
Genome-scale analysis of protein functions for *Sphingopyxis lindanitolerans* WS5A3p in context of *Sphingopyxis* pangenome. Bar chart illustrating protein clusters participation in different COGs functional categories for *Sphingopyxis* core pangenome (orange) and WS5A3p unique clusters (purple). (**A**) Cellular processes and signaling; (**B**) information storage and processing functions; (**C**) metabolism process; (**D**) unknown functions.

Next, we assigned representatives of core clusters to COGs. Nineteen percent and 20% of clusters were assigned to cellular processes and signaling (Figure 2A), and information storage and processing functions (Figure 2B), respectively. Almost 45% of all core clusters belonged to metabolic processes (Figure 2C). The enrichment in housekeeping functions like translation and ribosome biogenesis is clearly visible here, but amino acid and nucleotide metabolism were also very abundant processes.

We also identified and compared unique protein clusters for each genome included in the analysis (Table S2), and showed significant differences among the number of unique clusters; from four to 1667. The number of unique clusters positively correlate with phylogenetic distance to closest relatives (Spearman rank correlation *p*-value <2.2 × 10^−16^, Figure S3). As expected, closely related genomes contained fewer unique clusters (previously mentioned groups H1 and H2, but also genomes of Root154 and Root214) than more distant relatives.

In the pangenomic analysis, we found that the WS5A3p strain has 1274 unique protein clusters (one of the highest number among species analyzed in our dataset—Table S2), representing almost 31% of all clusters identified in its genome. Representatives of 1087 of unique protein clusters were successfully assigned to COGs, with 286 (26%) of them assigned to groups of general function prediction or unknown function (Figure 2D). However, within the general function group, we identified numerous aromatic ring-cleaving dioxygenases, monooxygenases, hydrolases and reductases that may participate in xenobiotic decomposition as well as members of the major facilitator superfamily (MFS) known to be involved in membrane transport. Here, we also found enzymes responsible for lindane turnover. Among poorly characterized COGs, the most abundant clusters were assigned to metabolic processes (369 clusters) and among them, 86 clusters were involved with inorganic ion transport and metabolism (Figure 2C). These clusters represent various iron-related proteins in high amounts, with the largest fraction as TonB-dependent receptors associated with the uptake and transport of large substrates such as iron siderophore complexes and vitamin B12 [58]. We also identified 123 clusters involved in transcription processes as well as clusters from replication, recombination and repair group (65 clusters) (Figure 2B). These were mainly transposases, integrases, and methyltransferases, indicating a high potential for recombinational processes. Analyzing the former, we detected transcriptional regulators assigned to numerous transcriptional regulator families like TerR, HxlR, GntR, LysR, IclR, AcrR, MarR, LuxR, ArsR, and AsnC.

For many *Sphingomonadaceae* members, a high number of genes putatively involved in aromatic compound degradation pathways have been annotated. Thus we also searched *Sphingopyxis* proteomes for the presence of proteins potentially involved in aromatic compound degradation. To reduce the number of calculations we selected H005 and H057 as representatives of H1 and H2 groups of nearly identical genomes respectively. We analyzed 31 proteomes and obtained 314 hits in total. The median number of identified proteins per proteome was 12 (IQR = 7), whereas the highest number of hits were found for *S. bauzanensis* DSM2271 (*n* = 38), strain LC363 (*n* = 29), *S. lindanitolerans* WS5A3p (*n* = 27) and strain H050 (*n* = 26) (Figure S4A,B). DSM2271 and WS5A3p strains were isolated from soils that were contaminated with either aromatic hydrocarbons or pesticides and might have acquired more genes related to the metabolism of aromatic compounds than other microorganisms analyzed. On the other hand, LC363 and H050 strains were isolated from water environments—cave and drinking water respectively—that are rather oligotrophic. For *S. lindanitolerans* WS5A3p we identified more enzymes potentially involved in aromatic compound degradation than for other strains isolated also from a γ-HCH contaminated environment like *S. flava* R11H (*n* = 15), *S. indica* DS15 (*n* = 9) and *S. terrae* subsp. *ummariensis* UI2 (*n* = 9). Majority of blastp hits (62%) were unique per genome, but also multiple copies of some proteins were identified (duplicates 31%, triplicates 4%, quadruplicates and more 2%). The most frequently identified proteins (that occurred more than 30 times) were dienelactone hydrolase (clCinC), 2-keto-4-pentanoate hydratase (CexF), 3-oxoadipate CoA-transferase (CinE), cytochrome P450 (CYP) and 4-hydroxy-2-oxovalerate aldolase (CexG). The highest frequency of the aforementioned enzymes can be explained by the fact that they conduct more distant reactions in their metabolic pathways and those substrates can participate in other pathways. The most frequently identified enzymes belonged to catechol 1,2-dioxygenation and catechol 2,3-dioxygenation route. Only for *S. bauzanensis* DSM2271 all seven proteins from catechol 2,3-dioxygenation route and only for strain KK2 all four proteins from catechol 1,2-dioxygenation route (for chlorocatechol) were identified (Figure S4C). Those results support the thesis that *Sphingopyxis*, like other sphingomonads, are specialized in the degradation of a particular compound rather than degrading a wide range of substrates [59]. Since the catechol is the main intermediate in the monoaromatic hydrocarbons biodegradation pathways, WS5Ap3 could be involved in degradation of benzene, phenol, benzoic acid or derivatives thereof. Catechol is also an intermediate product in the naphthalene and biphenyl pathways, so these compounds could also support growth of this strain, which may also confirm the presence of trans-o-hydroxybenzylidenepyruvate hydrolase-aldolase (NE), salicylaldehyde dehydrogenase (NF) (naphthalene-salicylate route) and biphenyl-2,3-diol 1,2-dioxygenase (BpC), 2,6-dioxo-6-phenylhexa-3-enoate hydrolase (BpD), benzolate 1,2-dioxygenase (BpG), dihydroxycyclohexadiene carboxylate dehydrogenase (BpH) (biphenyl-benzoate route) coding sequences in the genome (Figure S4C). However, the effect of individual aromatic compounds on the growth of this strain requires further study.

**Figure 3:**
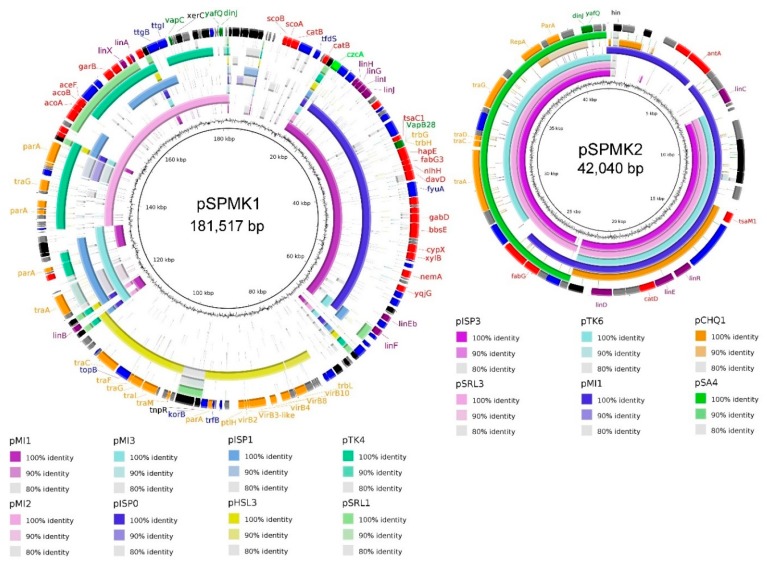
Comparative map of plasmid pSPMK1 and plasmid pSPMK2. Sequence similarity between pSPMK1 and pSPMK2 compared to selected plasmids derived from representative *Sphingomonadaceace*. The first ring represents GC content, rings 2–9 (for pSPMK1) and 2–7 (for pSPMK2) represents the similarity between chosen reference sequences. The last ring for both plasmids indicates open reading frames identified in the sequence, colored according to their function: black—transposases, integrases; purple—*lin* genes; orange—transfer, conjugation, partition genes; red—other catabolic genes; dark green—toxin/anti-toxin; light green—heavy metal resistance; blue—other genes, grey—hypothetical proteins.

Plasmids present in bacteria isolated from extreme environments often contain catabolic genes (e.g., for xenobiotic degradation) that are essential for survival in harsh conditions [60,61]. A thorough analysis of the WS5A3p genome led to the identification of two potential plasmid sequences. Their comparison to other known catabolic plasmid sequences derived from *Sphingomonadaceae* family is shown in Figure 3. Both of them have a mosaic structure of segments identified in other plasmids. The fact that the segments of high similarity are flanked by transposases suggests that they were acquired by fusion with ancestral plasmids [62]. The sequence of the larger plasmid—pSPMK1—has regions of high similarity to plasmids pMI1, pMI2 and pMI3 isolated from *Sphingobium* sp. MI1205—another γ-HCH degrader [59]. One region of pSPMK1 has exclusive similarity to plasmid pHSL3 from *Sphingomonas* sp. JJ-A5 (Genbank: CP018224.1), a chlorinated pesticide (alachlor) degrading bacterium. This region contains mostly conjugal transfer module, in particular, type IV secretion system genes. The shorter plasmid—pSPMK2—has the largest region similar to plasmids also derived from γ-HCH degrading microorganisms like pISP3 from *Sphingomonas* sp. MM-1 [63] and pSRL3 from *S. indicum* B90A [64,65]. Except for the first 8 kbp this region covers almost the entire sequence. Homologous regions include the stabilization and transfer module also present in pSA4 from non-γ-HCH-degrading strain *Novosphingobium resinovorum* [66]. The first 8 kbp of pSPMK2 is present only in the pMI1 plasmid (however in a different part of pMI1 than the sequence present in contig 1), and a small part in pCHQ1 from *S. japonicum* UT26S.

Since WS5A3p created clear zones on LB plates covered with γ-HCH and showed its degradation ability in a liquid medium, the genome sequence was analyzed for the presence of genes coding proteins involved in xenobiotic degradation pathways. pSPMK1 contains fragments of high similarity to the genes encoding a major pathway for the aerobic degradation of HCH isomers - the Lin pathway: *linA*, *linB*, *linX*, *linEb*, *linF*, and *linGHIJ* genes, whereas pSPMK2 contains *linC* and *linDER* genes (Table S3). Likely a consequence of numerous acquisitions, loss and structural rearrangements of mobile elements among different species, we can distinguish different localizations of *lin* genes. The location of *lin* genes ranges from almost solely chromosomally encoded in *S. japonicum* UT26S, to highly dispersed on multiple plasmids as in *Sphingobium* sp. MI1205 or MM-1 strains [62,67]. Numerous lines of evidence indicate that the accumulation of these genes is an ongoing process, as there are microorganisms identified with partial sets of *lin* genes in their genomes [68]. In *S. lindanitolerans* WS5A3p, the genes comprising the entire γ-HCH degradation pathway were detected exclusively on the two plasmids. The evolution and horizontal transfer of *lin* genes is connected with transposons [69]. By searching the IS FINDER database [70] we identified multiple insertion sequences (IS) in the WS5A3p genome, with IS*6* (*n* = 12) and IS*21* (*n* = 8) as the major IS families. The IS*6100* involved in the distribution of *linA* to *linF* genes in sphingomonad strains [12,42], belonging to the family of IS*6* insertion sequences were detected solely on pSPMK1 and pSPMK2 plasmids, not the chromosome (Figure S5). On pSPMK1 we identified seven complete IS*6100* and two partial matches on opposite edges of scaffold sequence, whereas on pSPMK2 only one complete IS*6100* and two partial matches. On pSPMK1 all of the *lin* genes are associated with at least one IS*6100* in close proximity. Arrangement of *linA* and *linX* genes resembles mostly the pattern detected in pMI2 where one of the flanking IS*6100* is localized distantly from *linA*. On the other hand, the *linB* gene is surrounded directly by IS*6100* sequences like on *Sphingobium* sp. TKS chromosome. Differently, than in other sphingomonad’s plasmids, *linC* gene is not flanked by two IS*6100* sequences [42]. In the case of pSPMK2, only partial IS*6100* sequence downstream *linC* gene was identified (Figure S5).

**Figure 4:**
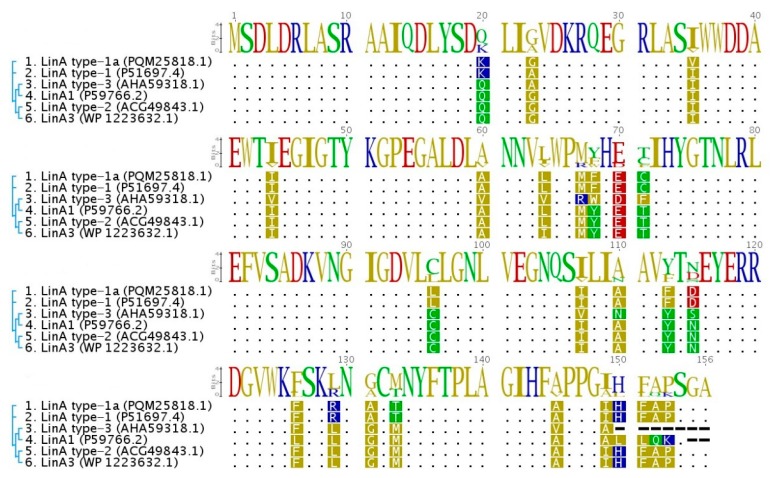
Multiple sequence alignment of LinA protein variants. The amino acid sequence of LinA type-1a from WS5A3p strain (1) compared to previously known variants of this protein (LinA type-1 from *Sphingobium japonicum* UT26 (2), LinA1 from *Sphingobium indicum* B90A (4), LinA3 from *Sphingobium sp.* HDIPO4 (6), LinA type-2 (5) and LinA type-3 from soil metagenomes (3)). Sequences were aligned and visualized using Geneious [27]. Dots correspond to the identical amino acids, dashes indicate the gaps. Phylogenetic relationships are indicated next to the sequence names and accession numbers.

Protein sequences of lindane upper degradation pathway proteins LinA and LinB are characterized by a high level of polymorphism and this alters their enantio- and stereoselectivity [71]. Sequences of Lin proteins identified in WS5A3p are almost identical to those from the archetypal strain UT26. The most differentiated in amino acid composition is an enzyme from the γ-HCH upper degradation pathway— LinA (named here LinA type-1a), which shows 98.7% identity with a LinA type 1 (also described as LinA2) [12] that was described to be more efficient in γ-HCH degradation than LinA1 from *S. indicum* B90A [72] to which it has a 90.3% identity (Figure 4). Also, three other variants of the LinA protein have been proposed in the literature [16,73,74,75]. To distinguish them, we use the nomenclature LinA3 (from *Sphingobium* sp. HDIPO4) and LinA type-2 and LinA type-3 (from the soil metagenomes). Their identities with WS5A3p LinA type-1a protein are 93.6% and 86.6%, respectively. LinA type-1a protein has conserved D25 and H73 residues necessary for its specific enzymatic activity. It differs from LinA type-1 and type-3 by A23G and I35V, which is unique for WS5A3p. While G23 is involved in protein thermostability [16], V35 have not been yet identified as accountable for specific protein features and need to be further investigated. Other LinA type-1a residues are the same as in LinA type-1. Based on the presence of K20, L96, and A131 residues we can predict that LinA type-1a will exhibit preference to the (−) enantiomers [76].

The genes encoding putative ABC transporter (*linKLMN*) necessary for γ-HCH utilization, reported for *Sphingobium* sp. were not identified in the WS5A3p genome. The highest amino acid identity of one of the WS5A3p ABC-transporter proteins to products of those genes were: 61% to LinK, 68% to LinL, 56% to LinM and 50% to LinN. The fact that homologs of the mentioned WS5A3p chromosomally encoded, putative ABC-transporter system are widely distributed among *Sphingopyxis* sp. with low identity levels (e.g., 42–75% identities at aa level for linKLMN identified in UT26 and MM-1 strains [62]) seems consistent with the hypothesis that several variants of these proteins underwent convergent evolution to facilitate lindane utilization [73] and more, this divergence roughly reflects the phylogenetic relationship between the strains [62].

## 4. Conclusions

In this work, we analyzed the genome of *Sphingopyxis lindanitolerans* WS5A3p derived from long-term pesticide-contaminated soil and capable of degrading xenobiotics. As predicted by its nucleotide sequence, WS5A3p degrades γ-HCH and may play an important role in the soil microbial community contaminated with this pesticide. This ability has been acquired by the process of horizontal gene transfer of *lin* genes. We also identified a new variant of LinA type-1 protein, namely LinA type-1a. This is the first well-documented communication of horizontal gene transfer of lindane degrading machinery to *Sphingopyxis* sp. Furthermore, a core protein set present in all sequenced representatives of this genus was described, as well as the unique protein sets identified for each genome, providing data for further investigations.

## Figures and Tables

**Table 1:** Gene counts associated with the general COGs (clusters of orthologous groups) functional categories for the genome of *Sphingopyxis lindanitolerans* WS5A3p, obtained from COGNIZER software [30].

Code	Description	WS5A3p	
		Value	%
J	Translation, ribosomal structure and biogenesis	241	6.8%
K	Transcription	287	8.0%
L	Replication, recombination and repair	319	8.9%
D	Cell cycle control, cell division, chromosome partitioning	38	1.1%
V	Defense mechanisms	81	2.3%
T	Signal transduction mechanisms	179	5.0%
M	Cell wall/membrane/envelope biogenesis	232	6.5%
N	Cell motility	57	1.6%
W	Extracellular structures	8	0.2%
U	Intracellular trafficking, secretion, and vesicular transport	209	5.9%
O	Posttranslational modification, protein turnover, chaperones	189	5.3%
C	Energy production and conversion	381	10.7%
G	Carbohydrate transport and metabolism	218	6.1%
E	Amino acid transport and metabolism	512	14.3%
F	Nucleotide transport and metabolism	91	2.5%
H	Coenzyme transport and metabolism	175	4.9%
I	Lipid transport and metabolism	481	13.5%
P	Inorganic ion transport and metabolism	481	13.5%
Q	Secondary metabolites biosynthesis, transport and catabolism	302	8.5%
R	General function prediction only	651	18.2%
S	Function unknown	277	7.8%

## Supplementary Materials

The following are available online at https://www.mdpi.com/2073-4425/10/9/688/s1, Figure S1: γ-HCH degrading abilities of *Sphingopyxis lindanitolerans* WS5A3p, Figure S2: Accumulation plots of the *Sphingopyxis pangenome*, Figure S3: Correlation between minimal phylogenetic distance to the closest neighbour and number of unique protein clusters in the genome, Figure S4: Identification of enzymes potentially involved in aromatic compound degradation, Figure S5: Organization of lin genes (blue) and IS6100 (green) identified on *Sphingopyxis lindanitolerans* WS5A3p plasmids, Table S1: General statistics of the *Sphingopyxis lindanitolerans* WS5A3p genome, Table S2: General information about 44 *Sphingopyxis* genomes used in this work, Table S3: Genome coordinates and locus tags of *lin* genes identified in WS5A3p genome sequence.

## References

[1] Nolan K., Kamrath J., Levitt J. (2012). Lindane toxicity: A comprehensive review of the medical literature. Pediatr. Dermatol..

[2] Vijgen J., Abhilash P.C., Li Y.F., Lal R., Forter M., Torres J., Singh N., Yunus M., Tian C., Schaffer A. (2011). Hexachlorocyclohexane (HCH) as new Stockholm Convention POPs—A global perspective on the management of Lindane and its waste isomers. Environ. Sci. Pollut. Res..

[3] Manickam N., Pathak A., Saini H.S., Mayilraj S., Shanker R. (2010). Metabolic profiles and phylogenetic diversity of microbial communities from chlorinated pesticides contaminated sites of different geographical habitats of India. J. Appl. Microbiol..

[4] Böltner D., Moreno-morillas S., Ramos J. (2005). 16S rDNA phylogeny and distribution of lin genes in novel hexachlorocyclohexane-degrading *Sphingomonas* strains. Environ. Microbiol..

[5] Verma H., Rani P., Singh A.K., Kumar R., Dwivedi V., Negi V., Lal R. (2015). *Sphingopyxis flava* sp. nov., isolated from a hexachlorocyclohexane (HCH)-contaminated soil. Int. J. Syst. Evolut. Microbiol..

[6] Jindal S., Dua A., Lal R. (2013). *Sphingopyxis indica* sp. nov., isolated from a high dose point hexachlorocyclohexane (HCH)- contaminated dumpsite. Int. J. Syst. Evolut. Microbiol..

[7] Sharma P., Verma M., Bala K., Nigam A., Lal R. (2010). *Sphingopyxis ummariensis* sp. nov., isolated from a hexachlorocyclohexane dump site. Int. J. Syst. Evolut. Microbiol..

[8] Feng G.D., Wang D.D., Yang S.Z., Li H.P., Zhu H.H. (2017). Genome-based reclassification of *Sphingopyxis ummariensis* as a later heterotypic synonym of *Sphingopyxis terrae*, with the descriptions of *Sphingopyxis terrae* subsp *terrae* subsp. nov. and *Sphingopyxis terrae* subsp. *ummariensis* subsp. nov.. Int. J. Syst. Evolut. Microbiol..

[9] Lal R., Dadhwal M., Kumari K., Sharma P., Singh A., Kumari H., Jit S., Gupta S.K., Nigam A., Lal D. (2008). *Pseudomonas* sp. to *Sphingobium indicum*: A journey of microbial degradation and bioremediation of Hexachlorocyclohexane. Indian J. Microbiol..

[10] Endo R., Kamakura M., Miyauchi K., Ohtsubo Y., Tsuda M., Fukuda M., Nagata Y.I. (2005). Identification and characterization of genes involved in the downstream degradation pathway of γ -hexachlorocyclohexane in *Sphingomonas paucimobilis* UT26. J. Bacteriol..

[11] Nagata Y., Endo R., Ito M., Ohtsubo Y., Tsuda M. (2007). Aerobic degradation of lindane (γ-hexachlorocyclohexane) in bacteria and its biochemical and molecular basis. Appl. Microbiol. Biotechnol..

[12] Imai R., Nagata Y., Fukuda M., Takagi M., Yano K. (1991). Molecular cloning of a *Pseudomonas paucimobilis* gene encoding a 17-kilodalton polypeptide that eliminates HCl molecules from γ-hexachlorocyclohexane. J. Bacteriol..

[13] Nagata Y., Futamura A., Miyauchi K., Takagi M. (1999). Two different types of dehalogenases, LinA and LinB, involved in γ- hexachlorocyclohexane degradation in *Sphingomonas paucimobilis* UT26 are localized in the periplasmic space without molecular processing. J. Bacteriol..

[14] Nagata Y., Hatta T., Imai R., Kimbara K., Fukuda M., Yano K., Takagi M. (1993). Purification and characterization of γ -hexachlorocyclohexane (γ -HCH) dehydrochlorinase (LinA) from *Pseudomonas paucimobilis*. Biosci. Biotechnol. Biochem..

[15] Nagata Y., Mori K., Takagi M., Murzin A.G., Damborsk J. (2001). Identification of protein fold and catalytic residues of γ-hexachlorocyclohexane dehydrochlorinase LinA. Proteins Struct. Funct. Genet..

[16] Macwan A.S., Kukshal V., Srivastava N., Javed S., Kumar A., Ramacheandran R. (2012). Crystal structure of the hexachlorocyclohexane dehydrochlorinase (LinA-Type2): Mutational analysis, thermostability and enantioselectivity. PLoS ONE.

[17] Okai M., Kubota K., Fukuda M., Nagata Y., Nagata K., Tanokura M. (2009). Crystallization and preliminary X-ray analysis of- hexachlorocyclohexane dehydrochlorinase LinA from *Sphingobium japonicum* UT26. Acta Crystallogr. Sect. F Struct. Biol. Cryst. Commun..

[18] Nagata Y., Nariya T., Ohtomo R., Fukuda M., Yano K., Takagi M. (1993). Cloning and sequencing of a dehalogenase gene encoding an enzyme with hydrolase γ-hexachlorocyclohexane involved in the degradation of y-hexachlorocyclohexane. J. Bacteriol..

[19] Kmunícek J., Hynková K., Jedlicka T., Nagata Y., Negri A., Gago F., Wade R.C., Damborsky J. (2005). Quantitative analysis of substrate specificity of haloalkane dehalogenase LinB from *Sphingomonas paucimobilis* UT26. Biochemistry.

[20] Nagata Y., Ohtomo R., Miyauchi K., Fukuda M., Yano K., Takagi M. (1994). Cloning and sequencing of a 2,5-dichloro-2,5-cyclohexadiene-1,4-diol dehydrogenase gene involved in the degradation of γ-hexachlorocyclohexane in *Pseudomonas paucimobilis*. J. Bacteriol..

[21] Miyauchi K., Suh S.K., Nagata Y., Takagi M. (1998). Cloning and sequencing of a 2,5-dichlorohydroquinone reductive dehalogenase gene whose product is involved in degradation of γ-hexachlorocyclohexane by *Sphingomonas paucimobilis*. J. Bacteriol..

[22] Miyauchi K., Adachi Y., Nagata Y. (1999). Cloning and sequencing of a novel meta-cleavage dioxygenase gene whose product is involved in degradation of γ -Hexachlorocyclohexane in *Sphingomonas paucimobilis*. J. Bacteriol..

[23] Kaminski M.A., Sobczak A., Spolnik G., Danikiewicz W., Dziembowski A., Lipinski L. (2018). *Sphingopyxis lindanitolerans* sp. nov. strain WS5A3pT enriched from a pesticide disposal site. Int. J. Syst. Evol. Microbiol..

[24] Martin M. (2011). Cutadapt removes adapter sequences from high-throughput sequencing reads. EMBnet. Journal.

[25] Joshi N., Fass J. (2011). Sickle: A sliding-window, adaptive, quality-based trimming tool for FastQ files [Internet]. https://github.com/najoshi/sickle.

[26] Bankevich A., Nurk S., Antipov D., Gurevich A., Dvorkin M., Kulikov A.S., Lesin V.M., Nikolenko S.I., Pham S., Prjibrlski A.D. (2012). SPAdes: A new genome assembly algorithm and its applications to single-cell sequencing. J. Comput. Biol..

[27] Kearse M., Moir R., Wilson A., Stones-Havas S., Cheung M., Sturrock S., Buxton S., Cooper A., Markowitz S., Duran C. (2012). Geneious Basic: An integrated and extendable desktop software platform for the organization and analysis of sequence data. Bioinformatics.

[28] Tatusova T., Dicuccio M., Badretdin A., Chetvernin V., Nawrocki P., Zaslavsky L., Lomsadze A., Pruitt K.D., Borodovsky M., OSTELL J. (2016). NCBI prokaryotic genome annotation pipeline. Nucleic Acid. Res..

[29] Petersen T.N., Brunak S., Heijne G.V., Nielsen H. (2011). SignalP 4.0: Discriminating signal peptides from transmembrane regions. Nat. Method.

[30] Bose T., Haque M.M., Reddy C., Mande S.S. (2015). COGNIZER: A framework for functional annotation of metagenomic datasets. PLoS ONE.

[31] Calteau A., Gachet M., Gautreau G., Josso A., Langlois J., Médigue C., Cruveiller S., Lajus A., Pereira H., Planel P. (2017). MicroScope-an integrated resource for community expertise of gene functions and comparative analysis of microbial genomic and metabolic data. Brief Bioinform..

[32] Blin K., Shaw S., Steinke K., Villebro R., Ziemert N., Lee S.Y., Medema M.H., Weber T. (2019). antiSMASH 5.0: Updates to the secondary metabolite genome mining pipeline. Nucleic Acids Res..

[33] Arndt D., Grant J.R., Marcu A., Sajed T., Pon A., Liang Y.J., Wishart D.S. (2016). PHASTER: A better, faster version of the PHAST phage search tool. Nucleic Acids Res..

[34] Grissa I., Vergnaud G., Pourcel C. (2007). CRISPRFinder: A web tool to identify clustered regularly interspaced short palindromic repeats. Nucleic Acids Res..

[35] Lauro F.M., Dougald D.M., Thomas T., Williams T.J., Egan S., Rice S., DeMaere M.Z., Ting L., Ertan H., Johnson J. (2009). The genomic basis of trophic strategy in marine bacteria. Proc. Natl. Acad. Sci.USA.

[36] Ohtsubo Y., Nonoyama S., Nagata Y., Numata M., Tsuchikane K., Hosoyama A., Yamazoe A., Tsuda M., Fujita N., Kawai F. (2016). Complete genome sequence of *Sphingopyxis terrae* strain 203-1 (NBRC 111660), a polyethylene glycol degrader. Genome Announc..

[37] Ohtsubo Y., Nagata Y., Numata M., Tsuchikane K., Hosoyama A., Yamazoe A., Tsuda M., Fujita N., Kawai F. (2015). Complete genome sequence of polyvinyl alcohol-degrading strain *Sphingopyxis* sp. 113P3 (NBRC 111507). Genome Announc..

[38] Gan H.Y., Gan H.M., Tarasco A.M., Busairi N.I., Barton H.A., Hudson A.O., Savka M.A. (2014). Whole-genome sequences of five oligotrophic bacteria isolated from deep within Lechuguilla Cave, New Mexico. Genome Announc..

[39] Okano K., Shimizu K., Maseda H., Kawauchi Y., Utsumi M., Itayama T., Zhang Z.Y., Sugiura N. (2015). Whole-genome sequence of the microcystin-degrading bacterium *Sphingopyxis* sp. strain C-1. Genome Announc..

[40] Kaminski M.A., Furmanczyk E.M., Dziembowski A., Sobczak A., Lipinski L. (2017). Draft genome sequence of the type strain *Sphingopyxis witflariensis* DSM 14551. Genome Announc..

[41] Kaminski M.A., Furmanczyk E.M., Dziembowski A., Sobczak A., Lipinski L. (2017). Draft genome sequence of the type strain *Sphingopyxis bauzanensis* DSM 22271. Genome Announc..

[42] Oelschlägel M., Rückert C., Kalinowski J., Schmidt G., Schlömann M., Tischler D. (2015). *Sphingopyxis fribergensis* sp. nov., a soil bacterium with the ability to degrade styrene and phenylacetic acid. Int. J. Syst. Evolut. Microbiol..

[43] García-Romero I., Pérez-Pulido A.J., González-Flores Y.E., Reyes-Ramírez F., Santero E., Floriano B. (2016). Genomic analysis of the nitrate-respiring *Sphingopyxis granuli* (formerly *Sphingomonas macrogoltabida*) strain TFA. BMC Genom..

[44] Ohtsubo Y., Nonoyama S., Nagata Y., Numata M., Tsuchikane K., Hosoyama A., Yamazoe A., Tsuda M., Fujita N., Kawai F. (2016). Complete genome sequence of *Sphingopyxis macrogoltabida* strain 203N (NBRC 111659), a polyethylene glycol degrader. Genome Announc..

[45] Pritchard L., Glover R.H., Humphris S., Elphinstone J.G., Toth I.K. (2016). Genomics and taxonomy in diagnostics for food security: Soft-rotting enterobacterial plant pathogens. Anal. Methods.

[46] Seemann T. (2014). Prokka: Rapid prokaryotic genome annotation. Bioinformatics..

[47] Segata N., Börnigen D., Morgan X.C., Huttenhower C. (2013). PhyloPhlAn is a new method for improved phylogenetic and taxonomic placement of microbes. Nat. Commun..

[48] Yu G., Smith D.K., Zhu H., Guan Y., Lam T.T.Y. (2017). Ggtree: An r package for visualization and annotation of phylogenetic trees with their covariates and other associated data. Methods Ecol. Evolut..

[49] Stamatakis A. (2014). RAxML Version 8: A tool for phylogenetic analysis and post-analysis of large phylogenies. Bioinformatics.

[50] Fu L., Niu B., Zhu Z., Wu S., Li W. (2012). CD-HIT: Accelerated for clustering the next-generation sequencing data. Bioinformatics.

[51] Kato H., Mori H., Maruyama F., Toyoda A., Oshima K., Endo R., Fuchu G., Miyakoshi M., Dozono A., Ohtsubo Y. (2015). Time-series metagenomic analysis reveals robustness of soil microbiome against chemical disturbance. DNA Res..

[52] Edgar R.C. (2004). MUSCLE: Multiple sequence alignment with high accuracy and high throughput. Nucleic Acids Res..

[53] Vernikos G., Medini D., Riley D.R., Tettelin H. (2015). Ten years of pan-genome analyses. Curr Opin Microbiol..

[54] Lefébure T., Stanhope M.J. (2007). Evolution of the core and pan-genome of *Streptococcus*: Positive selection, recombination, and genome composition. Genome Biol..

[55] Bottacini F., Medini D., Pavesi A., Turroni F., Foroni E., Riley D., Giubellini V., Tettelin H., Sinderen D.V., Ventura M. (2010). Comparative genomics of the genus *Bifidobacterium*. Microbiology.

[56] Parthasarathy S., Azam S., Lakshman Sagar A., Narasimha Rao V., Gudla R., Parapatla H., Yakkala H., Vemuri S.G., Siddavattam D. (2017). Genome-guided insights reveal organophosphate-degrading *Brevundimonas diminuta* as *Sphingopyxis wildii* and define its versatile metabolic capabilities and environmental adaptations. Genome Biol. Evolut..

[57] Fraser C.M., Gocayne J.D., White O., Adams M.D., Clayton R.A., Fleischmann R.D., Bult C.J., Kerlavage A.R., Sutton G., Kelley J.M. (1995). The minimal gene complement of *Mycoplasma genitalium*. Science.

[58] Koebnik R., Locher K.P., Van Gelder P. (2000). Structure and function of bacterial outer membrane proteins: Barrels in a nutshell. Mol. Microbiol..

[59] Tabata M., Ohhata S., Nikawadori Y., Sato T., Kishida K., Ohtsubo Y., Tsuda M., Nagata Y. (2016). Complete genome sequence of a γ-hexachlorocyclohexane-degrading bacterium, *Sphingobium* sp. strain MI1205. Genome Announc..

[60] Kumar S., Mukerji K.G., Lai R. (1996). Molecular aspects of pesticide degradation by microorganisms. Crit. Rev. Microbiol..

[61] Ramakrishnan B., Venkateswarlu K., Sethunathan N., Megharaj M. (2019). Local applications but global implications: Can pesticides drive microorganisms to develop antimicrobial resistance?. Sci. Total Environ..

[62] Tabata M., Ohhata S., Nikawadori Y., Kishida K., Sato T., Kawasumi T., Kato H., Ohtsubo Y., Tsuda M., Nagata Y. (2016). Comparison of the complete genome sequences of four γ-hexachlorocyclohexane-degrading bacterial strains: Insights into the evolution of bacteria able to degrade a recalcitrant man-made pesticide. DNA Res..

[63] Tabata M., Ohtsubo Y., Ohhata S., Tsuda M., Nagata Y. (2013). Complete genome sequence of the γ-hexachlorocyclohexane-degrading bacterium *Sphingomonas* sp. strain MM-1. Genome Announc..

[64] Anand S., Sangwan N., Lata P., Kaur J., Dua A., Singh A.K., Verma M., Kaur J., Khurana J.P., Khurana P. (2012). Genome sequence of *Sphingobium indicum* B90A, a hexachlorocyclohexane-degrading bacterium. J Bacteriol..

[65] Verma H., Bajaj A., Kumar R., Kaur J., Anand S., Nayyar N., Puri A., Singh Y., Khurana J.P., Lal R. (2017). Genome organization of *Sphingobium indicum* B90A: An archetypal hexachlorocyclohexane (HCH) degrading genotype. Genome Biol. Evolut..

[66] Hegedus B., Kos P.B., Balint B., Maroti G., Gan H.M., Perei K., Rákhely G. (2017). Complete genome sequence of *Novosphingobium resinovorum* SA1, a versatile xenobiotic-degrading bacterium capable of utilizing sulfanilic acid. J. Biotechnol..

[67] Tabata M., Endo R., Ito M., Ohtsubo Y., Kumar A., Tsuda M., Nagata Y. (2011). The *lin* genes for γ-hexachlorocyclohexane degradation in *Sphingomonas* sp. MM-1 proved to be dispersed across multiple plasmids. Biosci. Biotechnol. Biochem..

[68] Pearce S.L., Oakeshott J.G., Pandey G. (2015). Insights into ongoing evolution of the hexachlorocyclohexane catabolic pathway from comparative genomics of ten *Sphingomonadaceae* strains. G3 (Bethesda)..

[69] Dogra C., Raina V., Pal R., Suar M., Lal S., Gartemann K.H., Holliger C., Meer J.R.V.D., Lal R. (2004). Organization of lin genes and IS6100 among different strains of hexachlorocyclohexane-degrading *Sphingomonas paucimobilis*: Evidence for horizontal gene transfer. J. Bacteriol..

[70] Siguier P., Perochon J., Lestrade L., Mahillon J., Chandler M. (2006). ISfinder: The reference centre for bacterial insertion sequences. Nucleic Acids Res..

[71] Sharma P., Pandey R., Kumari K., Pandey G., Jackson C.J., Russell R.J., Oakeshott J.G., Lal R. (2011). Kinetic and sequence-structure-function analysis of known LinA variants with different hexachlorocyclohexane isomers. PLoS ONE..

[72] Kumari R., Subudhi S., Suar M., Dhingra G., Raina V., Dogra C., Lal S., Meer J.R.V.D., Holliger C., Lal R. (2002). Cloning and characterization of *lin* genes responsible for the degradation of hexachlorocyclohexane isomers by *Sphingomonas paucimobilis* strain B90. Appl. Environ Microbiol..

[73] Verma H., Kumar R., Oldach P., Sangwan N., Khurana J.P., Gilbert J.A., Lal R. (2014). Comparative genomic analysis of nine *Sphingobium* strains: Insights into their evolution and hexachlorocyclohexane (HCH) degradation pathways. BMC Genomics.

[74] Macwan A.S., Javed S., Kumar A. (2011). Isolation of a novel thermostable dehydrochlorinase (LinA) from a soil metagenome. 3Biotech.

[75] Shrivastava N., Prokop Z., Kumar A. (2015). Novel LinA type 3 δ-hexachlorocyclohexane dehydrochlorinase. Appl. Environ. Microbiol..

[76] Shrivastava N., Macwan A.S., Kohler H.P.E., Kumar A. (2017). Important amino acid residues of hexachlorocyclohexane dehydrochlorinases (LinA) for enantioselective transformation of hexachlorocyclohexane isomers. Biodegradation.

