# Supplementary material for: Genomic Analysis of γ-Hexachlorocyclohexane-Degrading Sphingopyxis lindanitolerans WS5A3p Strain in the Context of the Pangenome of Sphingopyxis
Source: Genes (Basel). 2019 Sep 6;10(9):688. doi: 10.3390/genes10090688 (PMC6771000; doi:10.3390/genes10090688)

SUPPLEMENTARY FILE

Title: Genomic analysis of γ-hexachlorocyclohexane degrading *Sphingopyxis lindanitolerans* WS5A3p strain in the context of the pangenome of *Sphingopyxis*

Michal A. Kaminski[1], Adam Sobczak[1,2], Andrzej Dziembowski[1,2], Leszek Lipinski[1,2,*]

[1]Institute of Biochemistry and Biophysics, Polish Academy of Sciences, Pawinskiego 5a, 02-106 Warsaw, Poland

[2]Institute of Genetics and Biotechnology, Faculty of Biology, University of Warsaw, Pawinskiego 5a, 02-106 Warsaw, Poland

* Correspondence: lechu@ibb.waw.pl (L.L.)

SUPPLEMENTARY TABLES AND FIGURES

Table S1. General statistics of the *Sphingopyxis lindanitolerans* WS5A3p genome.

Attribute	Chromosome	pSPMK1	pSPMK2	Total
Size (kbp)	4,149	181	42	4,372
DNA G + C (%)	65.3	62.8	62.6	65.1
Protein ORFs	3,812	180	49	4,041
tRNA genes	45	-	-	45
rRNA genes	3	-	-	3
ncRNA	2	-	-	2
tmRNA	1	-	-	1
Pseudo genes	68	20	4	92

Table S2. General information about 44 *Sphingopyxis* genomes used in this work. The number of genes as indicated in GenBank. The number of identified protein clusters was presented as the number of clusters identified by CD-HIT per genome. The number of unique protein clusters was presented as a protein cluster identified only in the analyzed genome. The asterisk symbol (*) indicates that for those genome groups unique clusters were counted as a sum of all groups representatives due to their high genome similarity.

**Organism**	**Country**	**Environment**	**GenBank assembly no.**	**Genome size (bp)**	**No. of genes**	**No. of identified protein clusters**	**No. of unique protein cluster s**
***S. alaskensis* RB2256**	USA	Seawater	GCA_000013985.1	3,373,713	3,287	3,236	573
***S. bauzanensis* DSM 22271**	Italy	Hydrocarbon-contaminated soil	GCA_002205675.1	4,258,005	4,149	4,097	1,207
***S. flava* R11H**	India	Hexachlorocyclohexane dumpsite soil	GCA_900168005.1	4,155,299	4,175	4,128	1,238
***S. fribergensis* Kp5.2**	Germany	Soil of meadow	GCA_000803645.1	5,202,172	4,938	4,886	1,035
***S. granuli* TFA**	Germany	Mud from Rhine river	GCA_001559015.1	4,679,853	4,350	4,297	1,549
***S. indica* DS15**	India	Hexachlorocyclohexane dumpsite soil	GCA_900188185.1	4,149,802	3,906	3,856	1,294
***S. macrogoltabida* 203**	Japan	Soil	GCA_001314325.1	5,748,623	5,572	5,506	1,667
***S. lindanitolerans* WS5A3p**	Poland	Hexachlorocyclohexane dumpsite soil	GCA_002993885.1	4,373,091	4,165	4,112	1,274
***Sphingopyxis* sp. 113P3**	Japan	Activated sludge	GCA_001278035.1	4,664,213	4,591	4,537	1,128
***Sphingopyxis* sp. A083**	USA	Drinking water	GCA_001468495.1	3,594,844	3,472	3,420	194
***Sphingopyxis* sp. C-1**	China	Lake water	GCA_001047015.1	4,583,092	4,324	4,272	604
***Sphingopyxis* sp. GW247-27LB**	USA	Ground Water	GCA_002277025.1	4,791,544	4,580	4,520	1,213
***Sphingopyxis* sp. H005**	USA	Drinking Water	GCA_001467395.1	5,103,573	4,832	4,780	25*
***Sphingopyxis* sp. H012**	USA	Drinking Water	GCA_001468235.1	5,056,279	4,786	4,734	

***Sphingopyxis* sp. H038**	USA	Drinking Water	GCA_001467405.1	5,058,140	4,790	4,739	
***Sphingopyxis* sp. H053**	USA	Drinking Water	GCA_001468225.1	5,130,000	4,862	4,811	
***Sphingopyxis* sp. H077**	USA	Drinking Water	GCA_001467445.1	5,256,605	4,980	4,929	
***Sphingopyxis* sp. H080**	USA	Drinking Water	GCA_001467455.1	5,194,868	4,925	4,874	
***Sphingopyxis* sp. H093**	USA	Drinking Water	GCA_001467475.1	5,346,988	5,083	5,032	
***Sphingopyxis* sp. H085**	USA	Drinking Water	GCA_001467385.1	5,221,350	4,966	4,915	
***Sphingopyxis* sp. H057**	USA	Drinking water	GCA_001468315.1	4,285,293	4,223	4,170	
***Sphingopyxis* sp. H067**	USA	Drinking water	GCA_001468345.1	4,308,725	4,235	4,182	
***Sphingopyxis* sp. H071**	USA	Drinking water	GCA_001468365.1	4,309,215	4,235	4,182	
***Sphingopyxis* sp. H073**	USA	Drinking water	GCA_001468395.1	4,309,020	4,236	4,183	34*
***Sphingopyxis* sp. H081**	USA	Drinking water	GCA_001468385.1	4,306,233	4,235	4,182	
***Sphingopyxis* sp. H100**	USA	Drinking water	GCA_001468425.1	4,306,674	4,236	4,183	
***Sphingopyxis* sp. H107**	USA	Drinking water	GCA_001468475.1	4,308,137	4,244	4,191	
***Sphingopyxis* sp. H050**	USA	Drinking water	GCA_001468265.1	4,363,228	4,198	4,145	768
***Sphingopyxis* sp. H115**	USA	Drinking water	GCA_001468465.1	4,493,891	4,324	4,274	638
***Sphingopyxis* sp. HIX**	USA	Drinking water	GCA_001468285.1	4,866,477	4,561	4,503	19
***Sphingopyxis* sp. HXXIV**	USA	Drinking water	GCA_001468305.1	4,885,113	4,591	4,533	23
***Sphingopyxis* sp. KK2**	USA	Lake water	GCA_001990265.1	4,336,942	4,224	4,167	871
***Sphingopyxis* sp. LC363**	USA	Cave water	GCA_000756385.1	4,210,757	3,958	3,906	424
***Sphingopyxis* sp. LC81**	USA	Cave water	GCA_000756375.1	4,397,290	4,158	4,109	488
***Sphingopyxis* sp. MC1**	USA	Waste water treatment plant	GCA_000371385.1	3,653,464	3,533	3,481	291
***Sphingopyxis* sp. P1IMeth2**	Australia	Coal seam gas water treatment pond	GCA_900185685.1	3,886,209	3,722	3,669	193
***Sphingopyxis* sp. QXT-31**	China	Surface soil of a Manganese mine	GCA_001984035.1	4,285,026	4,094	4,038	398
***Sphingopyxis* sp. Root1497**	Germany	*Arabidopsis thaliana* roots	GCA_001427085.1	4,801,179	4,537	4,478	957
***Sphingopyxis* sp. Root154**	Germany	*Arabidopsis thaliana* roots	GCA_001427105.1	4,726,935	4,442	4,388	4
***Sphingopyxis* sp. Root214**	Germany	*Arabidopsis thaliana* roots	GCA_001429105.1	4,724,829	4,440	4,386	5
***Sphingopyxis* sp. YR583**	Missing data	Populus sp. roots	GCA_900108295.1	4,312,099	4,060	4,003	708
***S. terrae* NBRC 15098**	Japan	Activated sludge	GCA_001598815.1	4,082,958	3,840	3,788	203
***S. ummariensis* UI2 (currently *S. terrae subsp. ummariensis* UI2)**	India	Hexachlorocyclohexane contaminated soil	GCA_900177755.1	3,577,011	3,469	3,413	330
***S. witflariensis* DSM 14551**	Germany	Activated sludge	GCA_002205635.1	4,306,761	4,207	4,149	1,303

Table S3. Genome coordinates and locus tags of *lin* genes identified in WS5A3p genome sequence.

Sequence Accession No.	Gene Name	Start	End	Length (nt)	Direction	Locus Tag	Genomic localization	Nucleotide identity (%) to that of UT26
NZ_PHFW01000004	linA	165,806	166,276	471	forward	CVO77_RS20540	Plasmid pSPMK1	99 (469/471)
NZ_PHFW01000004	linB	117,482	118,372	891	reverse	CVO77_RS20265	Plasmid pSPMK1	100 (891/891)
NZ_PHFW01000005	linC	6,750	7,502	753	reverse	CVO77_RS20705	Plasmid pSPMK2	100 (753/753)
NZ_PHFW01000005	linD	20,869	21,909	1,041	forward	CVO77_RS20770	Plasmid pSPMK2	100 (1,041/1,041)
NZ_PHFW01000005	linE	17,656	18,621	966	forward	CVO77_RS20750	Plasmid pSPMK2	100 (966/966)
NZ_PHFW01000004	linEb	61,081	62,043	963	reverse	CVO77_RS19960	Plasmid pSPMK1	99 (961/963)
NZ_PHFW01000004	linF	63,591	64,649	1,059	forward	CVO77_RS19970	Plasmid pSPMK1	99 (1,049/1,059)
NZ_PHFW01000004	linG	22,007	22,726	720	reverse	CVO77_RS19780	Plasmid pSPMK1	99 (719/720)
NZ_PHFW01000004	linH	21,369	22,007	639	reverse	CVO77_RS19775	Plasmid pSPMK1	99 (637/639)
NZ_PHFW01000004	linI	22,797	23,594	798	forward	CVO77_RS19785	Plasmid pSPMK1	99 (792/798)
NZ_PHFW01000004	linJ	23,630	24,841	1,212	forward	CVO77_RS19790	Plasmid pSPMK1	99 (1,202/1,212)
NZ_PHFW01000005	linR	16,613	17,524	912	reverse	CVO77_RS20745	Plasmid pSPMK2	100 (912/912)
NZ_PHFW01000004	linX	163,971	164,723	753	forward	CVO77_RS20525	Plasmid pSPMK1	100 (753/753)

Figure S1. γ-HCH degrading abilities of *Sphingopyxis lindanitolerans* WS5A3p. (A) Image of WS5A3p colonies grown on MSM solid medium with γ-HCH as a sole carbon source at 23°C for 14 days (colonies indicated by black arrow). The clear zone around the colony is visible. (B) Preliminary results of γ-HCH degradation in a liquid medium. Bacteria grown on MSM solid medium with γ-HCH for 5 days were used to inoculate 10x diluted LB medium with γ-HCH in concentration of 50 mg/L until they reached OD ~ 1. Next bacteria were washed 3 times with MSM liquid medium and resuspended in 50 mL of liquid MSM with γ-HCH in concentration of 50 mg/L. 10 ml of medium was extracted after inoculation (day 0), 3 and 7 days of incubation. Extraction was performed with ethyl acetate and the organic phase was analyzed using GC-MS for γ-HCH concentration.

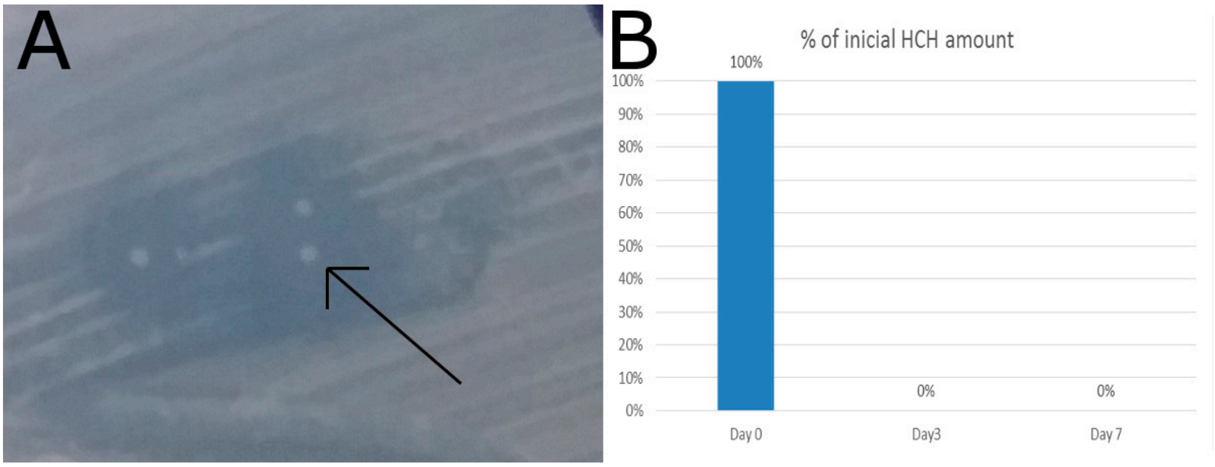

Figure S2. Accumulation plots of the *Sphingopyxis* pangenome. Illustrated values were calculated as the number of maximum possible permutations with the upper limit set to 10000. (A) Accumulation plot calculated for core pangenome size represented as the number of common protein clusters between the specified number of genomes. (B) Accumulation plot for pangenome size represented as the number of unique clusters between the specified number of genomes. Error bars represent standard deviations.

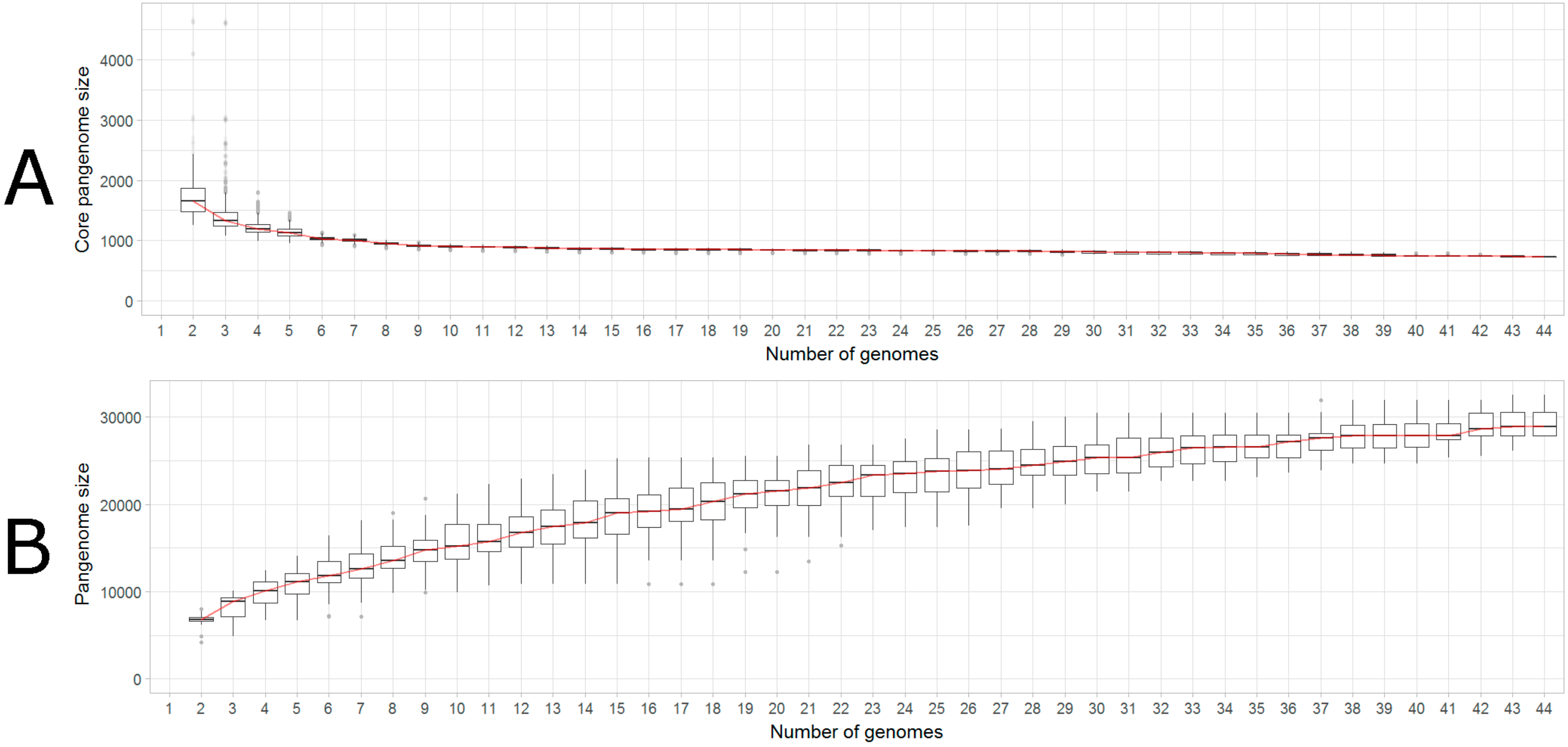

Figure S3. Correlation between minimal phylogenetic distance to the closest neighbor and the number of unique protein clusters in the genome. Each dot represents a separate genome. Blue line illustrates the fitted linear regression model. K-means clustering performed on analyzed dataset showed that it can be split into 4 distinct clusters. Genomes clustered together are represented by the same color.

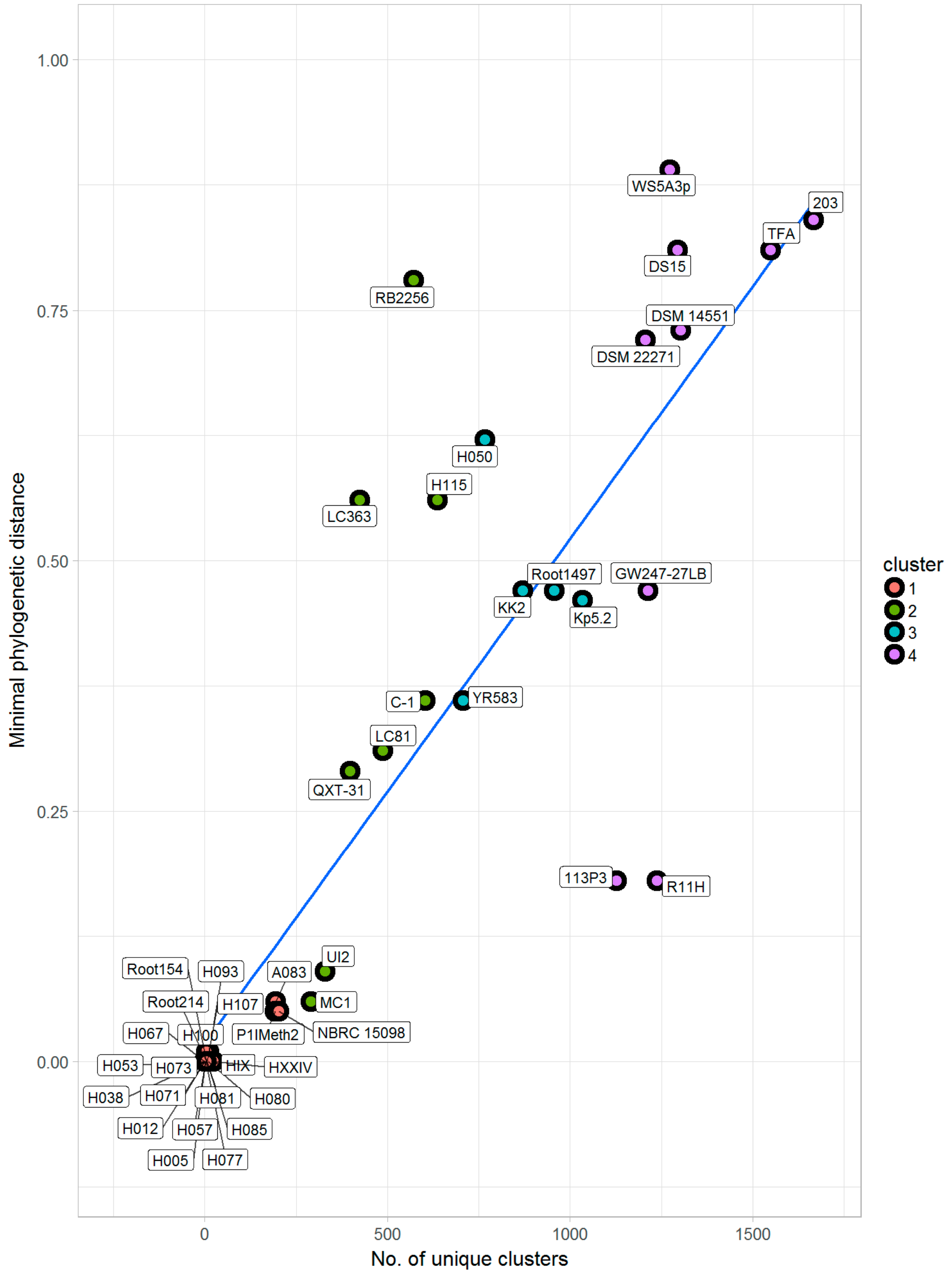

Figure S4. Identification of enzymes potentially involved in aromatic compound degradation. (A) Amount of protein hits per genome against the database of reference protein set. Bars colored by number of hits. (B) Distribution of amount of identified protein hits in all analyzed strains. (C) Amounts of specific enzyme hits per genome according to the metabolic route they belong to. Dots are colored by the metabolic route the protein belongs to and its size resembles number of identified hits per enzyme. Enzyme abbreviations from horizontal axis as described in Kato H. et al. 2015 [31].

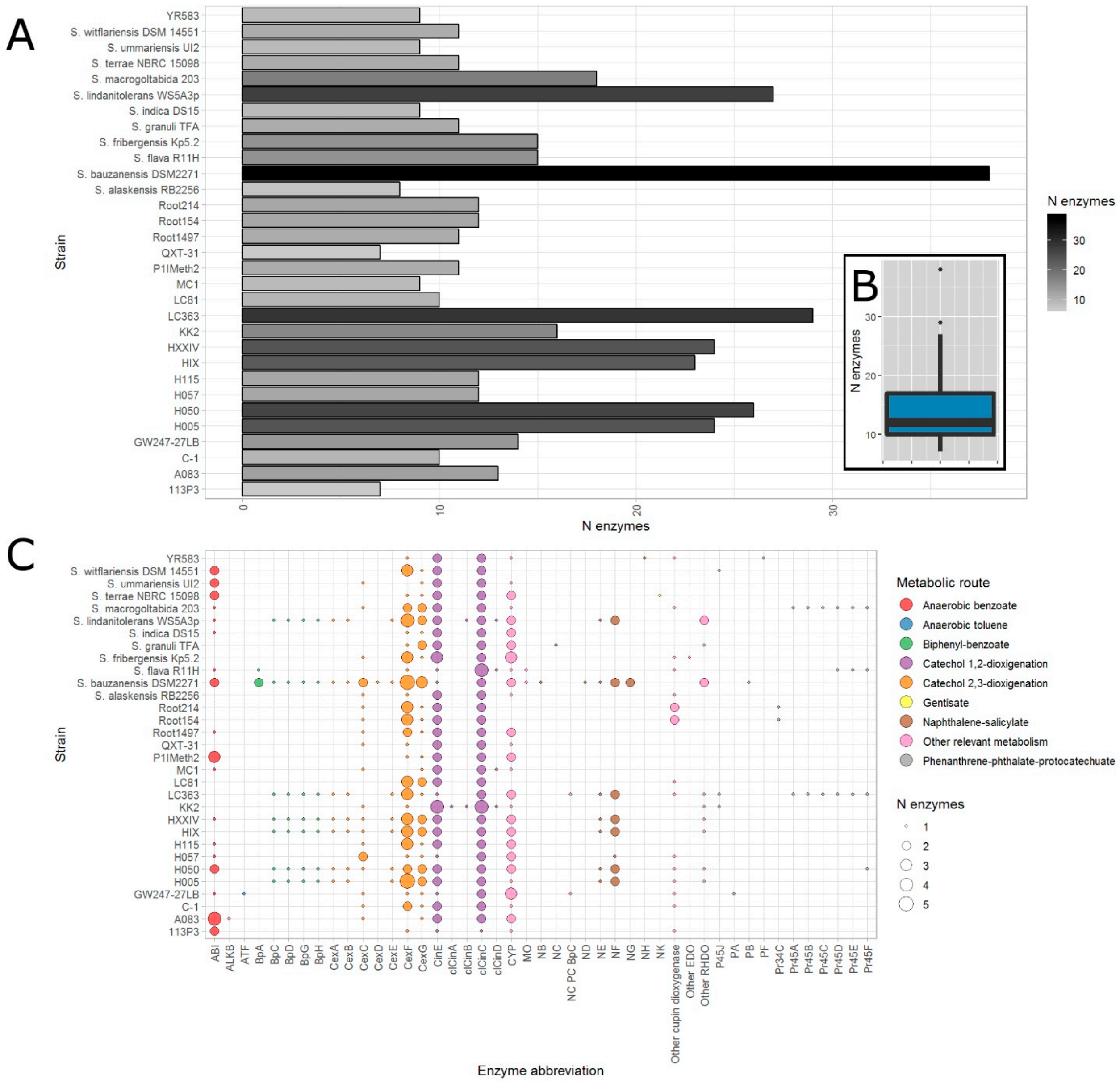

Figure S5. Organization of lin genes (blue) and IS*6100* (green) identified on *Sphingopyxis lindanitolerans* WS5A3p plasmids.

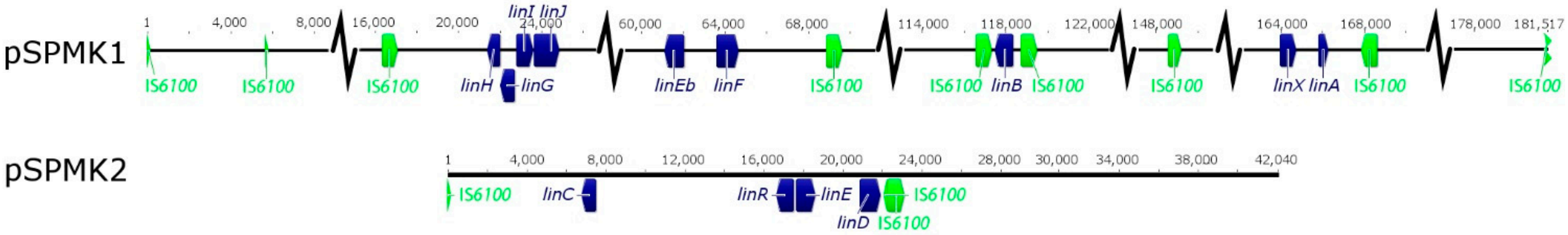

Supplement: Supplementary file 1 [file genes-10-00688-s001.pdf]
